# Supplementary figures and images for: The Effects of Topiroxostat, a Selective Xanthine Oxidoreductase Inhibitor, on Arterial Stiffness in Hyperuricemic Patients with Liver Dysfunction: A Sub-Analysis of the BEYOND-UA Study
Source: Biomedicines. 2023 Feb 23;11(3):674. doi: 10.3390/biomedicines11030674 (PMC10045538; doi:10.3390/biomedicines11030674)

**Figure S1.** Flowchart describing the sub-analysis.

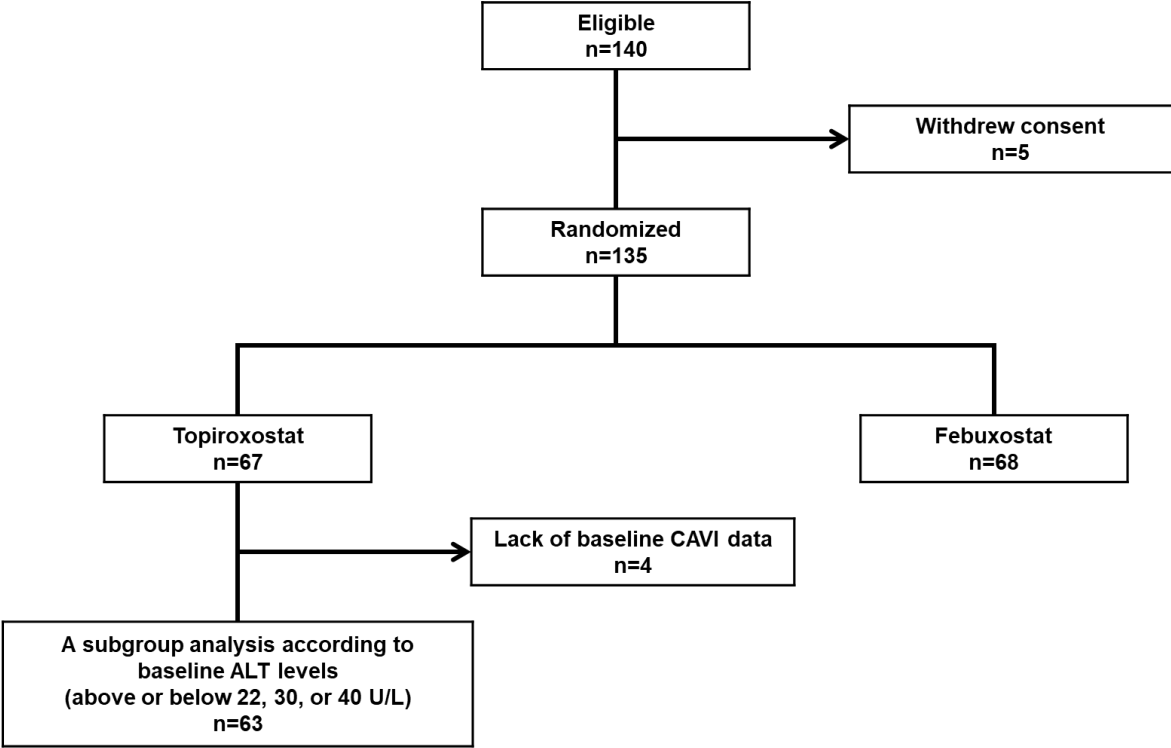

Supplement: Supplementary file 1 [file biomedicines-11-00674-s001.zip › biomedicines-2208092-supplementary.pdf]
